# An efficient dynamic ID-based remote user authentication scheme using self-certified public keys for multi-server environments

**DOI:** 10.1371/journal.pone.0202657

**Published:** 2018-10-09

**Authors:** Shudong Li, Xiaobo Wu, Dawei Zhao, Aiping Li, Zhihong Tian, Xiaodong Yang

**Affiliations:** 1 Cyberspace Institute of Advanced Technology, Guangzhou University, Guangzhou, China; 2 College of Computer, National University of Defense Technology, Hunan Changsha, China; 3 School of Software Engineering, Yantai Vocational College, Shandong Yantai, China; 4 Shandong Provincial Key Laboratory of Computer Networks, Shandong Computer Science Center (National Supercomputer Center in Jinan), Qilu University of Technology (Shandong Academy of Sciences), Jinan, China; 5 College of Computer Science and Engineering, Northwest Normal University, Gansu Lanzhou, China; Victoria University, AUSTRALIA

## Abstract

Recently, Li et al. proposed a novel smart card and dynamic ID-based remote user authentication scheme for multi-server environments. They claimed that their scheme can resist several types of attacks. However, through careful analysis, we find that Li et al.’s scheme is vulnerable to stolen smart card and off-line dictionary attacks, replay attacks, impersonation attacks and server spoofing attacks. By analyzing other similar schemes, we find that a certain type of dynamic ID-based multi-server authentication scheme in which only hash functions are used and whereby no registration center participates in the authentication and session key agreement phase faces difficulties in providing perfectly efficient and secure authentication. To compensate for these shortcomings, we propose a novel dynamic ID-based remote user authentication scheme for multi-server environments based on pairing and self-certified public keys. Security and performance analyses show that the proposed scheme is secure against various attacks and has many excellent features.

## Introduction

With the rapid development of network technologies, increasingly more people are beginning to use networks to acquire various services such as on-line financial information, on-line medical information, on-line shopping, on-line bill payment, and on-line documentation and data exchange. In addition, the architecture of servers providing services to be accessed over a network often consists of many different servers around the world instead of just one. Although they currently enjoy the comfort and convenience of the internet, people are facing emerging challenges with regard to network security.

Identity authentication is the key security issue facing various types of on-line applications and service systems. Before a user accesses services provided by a service provider server, mutual identity authentication between the user and server is needed to prevent unauthorized personnel from accessing services provided by the server and avoiding an illegal system defrauding the user by masquerading as a legitimate server. In a single-server environment, password-based authentication schemes [1] and enhanced versions that additionally use smart cards [2–9] are widely used to provide mutual authentication between the users and servers. However, conventional password-based authentication methods are not suitable for multi-server environments since each user need to not only log into various remote servers repetitively but also remember many different sets of identities and passwords if he/she wants to access these service provider servers. To resolve this problem, in 2000, based on the difficulty of factorization and hash functions, Lee and Chang [10] proposed a user identification and key distribution scheme that can be applied to multi-server environments. Since then, authentication schemes for multi-server environments have been widely investigated and designed by many researchers [11–37].

Based on the utilized basic cryptographic algorithms, multi-server authentication schemes can be divided into two types: hash-based authentication schemes and public-key-based authentication schemes. Simultaneously, among existing multi-server authentication schemes, some of them need a registration center (RC) to participate in the authentication and session key agreement phase, whereas others do not have this requirement. Therefore, based on whether the RC participates in the authentication and session key agreement phase, we divide the multi-server authentication schemes into RC-dependent authentication schemes and non-RC-dependent authentication schemes.

In this paper, we analyze a novel multi-server authentication scheme, Li et al.’s scheme [20], which is only based on hash functions and a non-RC-dependent authentication scheme. We find that this scheme is vulnerable to stolen smart cards and offline dictionary attacks, replay attacks, impersonation attacks and server spoofing attacks. By analyzing other similar schemes [15, 17–19], we find that the type of dynamic ID-based multi-server authentication scheme that only uses hash functions and are not dependent on RCs face difficulties in providing perfectly efficient and secure authentication. To compensate for these shortcomings, we propose a novel dynamic ID-based remote user authentication scheme for multi-server environments. Compared with previous related works, our scheme has many advantages. First, the scheme enjoys important security attributes, including being able to prevent various attacks, user anonymity, a lack of verification table, and local password verification. Second, the scheme does not use a timestamp; therefore, it avoids the clock synchronization problem. Further, the scheme uses self-certified public keys, by which the user’s public key can be computed directly from the signature of the trusted third party on the user’s identity instead of verifying the public key using an explicit signature on a user’s public key. Therefore, our scheme is more practical and universal for multi-server environments. Finally, the performance and cost analysis show that our scheme is very efficient and more secure than other related schemes.

## Related works

A large number of authentication schemes have been proposed for multi-server environments. Hash functions are a key technology in the construction of multi-server authentication schemes. In 2004, Juang et al. [11] proposed an efficient multi-server password authenticated key agreement scheme based on a hash function and symmetric key cryptosystem. In 2009, Hsiang and Shih [12] proposed a dynamic ID-based remote user authentication scheme for multi-server environments in which only a hash function is used. However, Sood et al. [13] found that Hsiang and Shih’s scheme is susceptible to replay attacks, impersonation attacks and stolen smart card attacks. Moreover, the password change phase of Hsiang and Shih’s scheme is insecure. Later, Sood et al. presented a novel dynamic identity-based authentication protocol for multi-server architectures to resolve the security flaws of Hsiang and Shih’s scheme [13]. In addition, Sood et al.’s protocol is practical and computationally efficient because only nonce, one-way hash functions and XOR operations are used in its implementation. After that, Li et al. [14] noted that Sood et al.’s protocol remains vulnerable to leak-of-verifier attacks, stolen smart card attacks and impersonation attacks. Simultaneously, Li et al. [14] proposed another dynamic identity-based authentication protocol for multi-server architectures. However, the above-mentioned schemes are all RC-dependent multi-server authentication schemes. In 2009, Liao and Wang [15] proposed a dynamic ID-based multi-server authentication scheme that is based on hash functions and does not depend on RCs. This scheme not only satisfies all requirements for multi-server environments but also achieves efficient computation. However, Liao and Wang’s scheme has been found to be vulnerable to insider attacks, masquerade attacks, server spoofing attacks, and registration center spoofing attacks and is not reparable [16]. Later, Shao et al. [17] and Lee et al. [18, 19] proposed similar types of multi-server authentication schemes. In 2012, Li et al. [20] noted that Lee et al.’s scheme [18] cannot withstand forgery attacks or server spoofing attacks and cannot provide proper authentication; they then proposed a novel dynamic ID-based multi-server authentication scheme that only uses a hash function and is not dependent on RCs. Moreover, the scheme is found to be suitable for financial security authentication. However, through careful analysis, we find that Li et al.’s scheme [20] remains vulnerable to stolen smart card and offline dictionary attacks, replay attacks, impersonation attacks and server spoofing attacks. We also analyzed Shao et al.’s scheme [17] and Lee et al.’s scheme [19]; they are all vulnerable to stolen smart card and offline dictionary attacks, replay attacks, impersonation attacks and server spoofing attacks. In general, it is difficult to construct a secure dynamic ID-based and non-RC-dependent multi-server authentication scheme if only hash functions are used.

Public-key cryptography is another useful technique that is widely used in the construction of multi-server authentication schemes. In 2000, Lee and Chang [21] proposed a user identification and key distribution scheme in which the difficulty of factorization on public key cryptography is used. In 2001, Tsaur [22] proposed a remote user authentication scheme based on an RSA cryptosystem and Lagrange interpolating polynomials for multi-server environments. Then, Lin et al. [23] proposed a multi-server authentication protocol based on the simple geometric properties of the Euclidean and discrete logarithm problem concept. In their scheme, the system does not need to maintain a verification table, and the users who have registered with the servers do not need to remember different login passwords for various servers. Since traditional public key cryptographic algorithms require many expensive computations and consume substantial energy, Geng and Zhang [24] proposed a dynamic ID-based user authentication and key agreement scheme for multi-server environments using bilinear pairings. However, Geng and Zhang’s scheme cannot withstand user spoofing attacks [25]. Later, Tseng et al. [26] proposed an efficient pairing-based user authentication scheme with smart cards. Performance analysis and experimental data demonstrate that their scheme is well suited for mobile devices with limited computing capabilities. However, in 2013, Liao and Hsiao [27] noted that Tseng et al.’s scheme is vulnerable to insider attacks, offline dictionary attacks and malicious server attacks and cannot provide proper mutual authentication and session key agreement. Simultaneously, Liao and Hsiao proposed a novel non-RC-dependent multi-server remote user authentication scheme using self-certified public keys for mobile clients [27]. Recently, Chou et al. [28] found that Liao and Hsiao’s scheme cannot withstand password guessing attacks. Furthermore, through careful analysis, we found that Liao and Hsiao’s scheme remains vulnerable to denial of service attacks and cannot ensure a user’s anonymity or provide local password verification. In this paper, we propose a secure dynamic ID-based and non-RC-dependent multi-server authentication scheme using pairing and self-certified public keys.

## Preliminaries

In this section, we introduce the concepts of bilinear pairings, self-certified public keys, as well as some related mathematical assumptions.

### Bilinear pairings

Let *G*_1_ be an additive cyclic group with a large prime order *q*, and let *G*_2_ be a multiplicative cyclic group with the same order *q*. In particular, *G*_1_ is a subgroup of the group of points on an elliptic curve over a finite field *E*(*F*_*p*_), and *G*_2_ is a subgroup of the multiplicative group over a finite field. *P* is a generator of *G*_1_.

A bilinear pairing is a map *e*: *G*_1_ × *G*_1_ → *G*_2_ and satisfies the following properties:

(1) Bilinear: *e*(*aP*, *bQ*) = *e*(*P*, *Q*)^*ab*^ for all *P*, *Q* ∈ *G*_1_ and $a,b\in Z_{q}^{*}$.

(2) Non-degenerate: There exists *P*, *Q* ∈ *G*_1_ such that *e*(*P*, *Q*)≠1.

(3) Computability: There is an efficient algorithm to compute *e*(*P*, *Q*) for all *P*, *Q* ∈ *G*_1_.

### Self-certified public keys

In [27], Liao et al. first proposed a key distribution scheme based on self-certified public keys (SCPKs) [38, 39] among the service servers. Using the SCPK, a user’s public key can be computed directly from the signature of the trusted third party (TTP) on the user’s identity instead of verifying the public key using an explicit signature on a user’s public key. The SCPK scheme is described as follows.

(1) Initialization: The trusted third party (TTP) first generates all the needed parameters of the scheme. The TTP chooses a non-singular high elliptic curve *E*(*F*_*p*_) defined over a finite field, which is used with a point-based generator *P* of prime order *q*. Then, the TTP freely chooses his/her secret key *s*_*T*_ and computes his/her public key *pub*_*T*_ = *s*_*T*_ ⋅ *P*. The related parameters and *pub*_*T*_ are publicly and authentically available.

(2) Private key generation: A user *A* chooses a random number *k*_*A*_, computes *K*_*A*_ = *k*_*A*_ ⋅ *P* and sends his/her identity *ID*_*A*_ and *K*_*A*_ to the TTP. The TTP chooses a random number *r*_*A*_, computes *W*_*A*_ = *K*_*A*_ + *r*_*A*_ ⋅ *P* and ${\bar{s}}_{A}=s_{T}\cdot h\left(ID_{A}\|W_{A}\right)+r_{A}$, and sends *W*_*A*_ and ${\bar{s}}_{A}$ to user *A*. Then, *A* obtains his/her secret key by calculating $s_{A}={\bar{s}}_{A}+k_{A}$.

(3) Public key extraction: Anyone can calculate *A*’s public key *pub*_*A*_ = *h*(*ID*_*A*_ ∥ *W*_*A*_)*pub*_*T*_ + *W*_*A*_ given *W*_*A*_.

### Related mathematical assumptions

To prove the security of our proposed protocol, we present some important mathematical problems and assumptions for bilinear pairings defined on elliptic curves. The related concrete description can be found in [40, 41].

(1) Computational discrete logarithm (CDL) problem: Given *R* = *x* ⋅ *P*, where *P*, *R* ∈ *G*_1_, it is easy to calculate *R* given *x* and *P*, but it is hard to determine *x* given *P* and *R*.

(2) Elliptic curve factorization (ECF) problem: Given two points *P* and *R* = *x* ⋅ *P* + *y* ⋅ *P* for $x,y\in Z_{q}^{*}$, it is hard to find *x* ⋅ *P* and *y* ⋅ *P*.

(3) Computational Diffie-Hellman (CDH) problem: Given *P*, *xP*, *yP* ∈ *G*_1_, it is hard to compute *xyP* ∈ *G*_1_.

## Review and cryptanalysis of Li et al.’s authentication scheme

### Review of Li et al.’s scheme

There are three participants in Li et al.’s scheme: the registration center *RC*, the server *S*_*j*_, and the user *U*_*i*_. *RC* generates the master secret key *x* and a secret number *y* to construct *h*(*x*‖*y*) and *h*(*SID*_*j*_‖*h*(*y*)), in which *SID*_*j*_ is the identity of server *S*_*j*_; then, it delivers them to the server *S*_*j*_ through a secure channel. Li et al.’s scheme contains four phases:the registration phase, the login phase, the verification phase and the password change phase.

#### Registration phase

When the remote user authentication scheme starts, the registration process should be first performed by the user *U*_*i*_ and *RC*:

(1) *U*_*i*_ generates a random number *b* and freely chooses his/her identity *ID*_*i*_ and the password *PW*_*i*_. Then, *U*_*i*_ calculates *A*_*i*_ = *h*(*b* ⊕ *PW*_*i*_). After that, *U*_*i*_ transmits *ID*_*i*_ and *A*_*i*_ to *RC* for registration through a secure channel.

(2) *RC* computes *B*_*i*_ = *h*(*ID*_*i*_‖*x*), *C*_*i*_ = *h*(*ID*_*i*_‖*h*(*y*)‖*A*_*i*_), *D*_*i*_ = *h*(*B*_*i*_‖*h*(*x*‖*y*)) and *E*_*i*_ = *B*_*i*_ ⊕ *h*(*x*‖*y*). Then, *RC* stores {*C*_*i*_, *D*_*i*_, *E*_*i*_, *h*(⋅), *h*(*y*)} on the smart card of *U*_*i*_ and sends it to *U*_*i*_ by a secure channel.

(3) *U*_*i*_ adds the random number *b* into the smart card, which ultimately possesses the information {*C*_*i*_, *D*_*i*_, *E*_*i*_, *b*, *h*(⋅), *h*(*y*)}.

#### Login phase

When user *U*_*i*_ wants to log into the server *S*_*j*_, the following procedures should be performed:

(1) After the smart card is inserted into the card reader, the user is prompted to enter his/her *ID*_*i*_ and *PW*_*i*_. After that, the smart card calculates *A*_*i*_ = *h*(*b* ⊕ *PW*_*i*_), $C_{i}^{*}=h(ID_{i}\left\|h\left(y\right)\right\|A_{i})$ and checks whether $C_{i}^{*}$ is equal to *C*_*i*_. If $C_{i}^{*}$ is equal to *C*_*i*_, the Login process continues. Otherwise, the session will be aborted.

(2) The smart card produces a number *N*_*i*_ randomly and calculates *P*_*ij*_ = *E*_*i*_ ⊕ *h*(*h*(*SID*_*j*_‖*h*(*y*))‖*N*_*i*_), *CID*_*i*_ = *A*_*i*_ ⊕ *h*(*D*_*i*_‖*SID*_*j*_‖*N*_*i*_), *M*_1_ = *h*(*P*_*ij*_‖*CID*_*i*_‖*D*_*i*_‖*N*_*i*_) and *M*_2_ = *h*(*SID*_*j*_‖*h*(*y*)) ⊕ *N*_*i*_.

(3) The smart card transmits the login request message {*P*_*ij*_, *CID*_*i*_, *M*_1_, *M*_2_} to *S*_*j*_.

#### Verification phase

When *S*_*j*_ receives the login request message, the mutual authentication and session key agreement between *S*_*j*_ and *U*_*i*_ will be performed in accordance with the following steps.

(1) The server *S*_*j*_ calculates *N*_*i*_ = *M*_2_ ⊕ *h*(*SID*_*j*_‖*h*(*y*)), *E*_*i*_ = *P*_*ij*_ ⊕ *h*(*h*(*SID*_*j*_‖*h*(*y*))‖*N*_*i*_), *B*_*i*_ = *E*_*i*_ ⊕ *h*(*x*‖*y*), *D*_*i*_ = *h*(*B*_*i*_‖*h*(*x*‖*y*)), and *A*_*i*_ = *CID*_*i*_ ⊕ *h*(*D*_*i*_‖*SID*_*j*_‖*N*_*i*_).

(2) The server *S*_*j*_ calculates *h*(*P*_*ij*_‖*CID*_*i*_‖*D*_*i*_‖*N*_*i*_); if the calculated result is not equal to *M*_1_, *S*_*j*_ rejects the login request and aborts this session. Otherwise, *S*_*j*_ accepts the login request message. Then, *S*_*j*_ chooses a random number *N*_*j*_ and calculates *M*_3_ = *h*(*D*_*i*_‖*A*_*i*_‖*N*_*j*_‖*SID*_*j*_), *M*_4_ = *A*_*i*_ ⊕ *N*_*i*_ ⊕ *N*_*j*_. Finally, *S*_*j*_ sends {*M*_3_, *M*_4_} to *U*_*i*_.

(3) According to the received message {*M*_3_, *M*_4_}, *U*_*i*_ calculates *N*_*j*_ = *A*_*i*_ ⊕ *N*_*i*_ ⊕ *M*_4_, $M_{3}^{*}=h(D_{i}\|A_{i}\|N_{j}\left\|SID_{j}\right)$ and verifies whether $M_{3}^{*}$ is equal to *M*_3_. If they are not equal, *U*_*i*_ rejects these messages and terminates this session. Otherwise, *U*_*i*_ successfully authenticates *S*_*j*_. In addition, *U*_*i*_ calculates *M*_5_ = *h*(*D*_*i*_‖*A*_*i*_‖*N*_*i*_‖*SID*_*j*_) and sends it to *S*_*j*_.

(4) The server *S*_*j*_ computes *h*(*D*_*i*_‖*A*_*i*_‖*N*_*i*_‖*SID*_*j*_) and compares it with the received {*M*_5_} sent from *U*_*i*_. If they are equal, *U*_*i*_ is successfully authenticated by *S*_*j*_, and the mutual authentication is completed. After the mutual authentication phase, the user *U*_*i*_ and the server *S*_*j*_ calculate *SK* = *h*(*D*_*i*_‖*A*_*i*_‖*N*_*i*_‖*N*_*j*_‖*SID*_*j*_) as their session key in future secure communication.

#### Password change phase

For security, the password of the user should be changed frequently. The password change phase is performed when user *U*_*i*_ wants to replace the old password *PW*_*i*_ with a new password $PW_{i}^{new}$.

(1) The user *U*_*i*_ inserts his/her smart card into the card reader and inputs his/her *ID*_*i*_ and *PW*_*i*_.

(2) The smart card calculates *A*_*i*_ = *h*(*b* ⊕ *PW*_*i*_), $C_{i}^{*}=h(ID_{i}\left\|h\left(y\right)\right\|A_{i})$ and verifies whether $C_{i}^{*}$ is equal to *C*_*i*_. If they are not equal, the password change request will be rejected. Otherwise, the user *U*_*i*_ provides a new random number *b*^*new*^ and a new password $PW_{i}^{new}$.

(3) The smart card calculates $A_{i}^{new}=h\left(b^{new}⊕PW_{i}^{new}\right)$ and $C_{i}^{new}=h(ID_{i}\left\|h\left(y\right)\right\|A_{i}^{new})$.

(4) The smart card uses $C_{i}^{new}$ and *b*^*new*^ to replace *C*_*i*_ and *b*. The password change phase is completed.

### Cryptanalysis of Li et al.’s scheme

Li et al. claimed that their scheme can resist many types of attacks and satisfy all the essential requirements for multi-server architecture authentication. However, if we assume that *A* is an adversary who has broken a user *U*_*m*_ and a server *S*_*n*_ or a combination of a malicious user *U*_*m*_ and a dishonest server *S*_*n*_, then *A* can obtain the secret number *h*(*x*‖*y*) and *h*(*y*) and perform stolen smart card and offline dictionary attacks, replay attacks, impersonation attacks and server spoofing attacks on Li et al.’s scheme. The concrete cryptanalysis of the Li et al.’s scheme is shown as follows.

#### Stolen smart card and offline dictionary attacks

If a user *U*_*i*_’s smart card is stolen by an adversary *A*, *A* can extract the information {*C*_*i*_, *D*_*i*_, *E*_*i*_, *b*, *h*(⋅), *h*(*y*)} from the memory of the stolen smart card. Furthermore, if *A* intercepts a valid login request message {*P*_*ij*_, *CID*_*i*_, *M*_1_, *M*_2_} sent from user *U*_*i*_ to server *S*_*j*_ in the public communication channel, *A* can compute *N*_*i*_ = *h*(*SID*_*j*_‖*h*(*y*)) ⊕ *M*_2_, *E*_*i*_ = *P*_*ij*_ ⊕ *h*(*h*(*SID*_*j*_‖*h*(*y*))‖*N*_*i*_), *B*_*i*_ = *E*_*i*_ ⊕ *h*(*x*‖*y*), *D*_*i*_ = *h*(*B*_*i*_‖*h*(*x*‖*y*)) and *A*_*i*_ = *CID*_*i*_ ⊕ *h*(*D*_*i*_‖*SID*_*j*_‖*N*_*i*_) using *h*(*y*) and *h*(*x*‖*y*). Then, *A* can launch an offline dictionary attack on *C*_*i*_ = *h*(*ID*_*i*_‖*h*(*y*)‖*A*_*i*_) to determine the identity *ID*_*i*_ of user *U*_*i*_ because *A* knows the values of *A*_*i*_ and *h*(*y*) corresponding to the user *U*_*i*_. In addition, *A* can launch offline dictionary attacks on *A*_*i*_ = *h*(*b* ⊕ *PW*_*i*_) to determine the password *PW*_*i*_ of *U*_*i*_ because *A* knows the value of *b* from the stolen smart card of the user *U*_*i*_. Now, *A* possesses the valid smart card of user *U*_*i*_, knows the identity *ID*_*i*_ and password *PW*_*i*_ corresponding to user *U*_*i*_ and hence can login to any service provider server.

#### Replay attacks

A replay attack is when an adversary replays the same message of a receiver or sender again. If adversary *A* has intercepted a valid login request message {*P*_*ij*_, *CID*_*i*_, *M*_1_, *M*_2_} sent from user *U*_*i*_ to server *S*_*j*_ in the public communication channel, then *A* can compute *N*_*i*_ = *h*(*SID*_*j*_‖*h*(*y*)) ⊕ *M*_2_, *E*_*i*_ = *P*_*ij*_ ⊕ *h*(*h*(*SID*_*j*_‖*h*(*y*))‖*N*_*i*_), *B*_*i*_ = *E*_*i*_ ⊕ *h*(*x*‖*y*), *D*_*i*_ = *h*(*B*_*i*_‖*h*(*x*‖*y*)) and *A*_*i*_ = *CID*_*i*_ ⊕ *h*(*D*_*i*_‖*SID*_*j*_‖*N*_*i*_) using *h*(*y*) and *h*(*x*‖*y*). Then, adversary *A* can replay this login request message {*P*_*ij*_, *CID*_*i*_, *M*_1_, *M*_2_} to *S*_*j*_ by masquerading as the user *U*_*i*_ at some later time. After verification of the login request message, *S*_*j*_ computes *M*_3_ = *h*(*D*_*i*_‖*A*_*i*_‖*N*_*j*_‖*SID*_*j*_) and *M*_4_ = *A*_*i*_ ⊕ *N*_*i*_ ⊕ *N*_*j*_ and sends the message {*M*_3_, *M*_4_} to *A*, who is masquerading as the user *U*_*i*_. The adversary *A* can verify the received value of {*M*_3_, *M*_4_} and compute $M_{5}^{\prime }=h(D_{i}\|A_{i}\|N_{i}\left\|SID_{j}\right)$ since they know the values of *N*_*i*_, *E*_*i*_, *B*_*i*_, *D*_*i*_ and *A*_*i*_. Then, *A* sends $\left\{M_{5}^{\prime }\right\}$ to the server *S*_*j*_. The server *S*_*j*_ computes *h*(*D*_*i*_‖*A*_*i*_‖*N*_*i*_‖*SID*_*j*_) and checks it with the received message $\left\{M_{5}^{\prime }\right\}$. This equivalency authenticates the legitimacy of the user *U*_*i*_ and the service provider server *S*_*j*_, and the login request is accepted. Finally, after mutual authentication, adversary *A* masquerading as the user *U*_*i*_ and the server *S*_*j*_ agree on the common session key as *SK* = *h*(*D*_*i*_‖*A*_*i*_‖*N*_*i*_‖*N*_*j*_‖*SID*_*j*_). Therefore, the adversary *A* can masquerade as user *U*_*i*_ to login to server *S*_*j*_ by replaying the same login request message that had been sent from *U*_*i*_ to *S*_*j*_.

#### Impersonation attacks

In this subsection, we show that an adversary *A* who possesses *h*(*y*) and *h*(*x*‖*y*) can masquerade as any user *U*_*i*_ to login to any server *S*_*j*_ as follows.

Adversary *A* chooses two random numbers *a*_*i*_ and *b*_*i*_ and computes *A*_*i*_ = *h*(*a*_*i*_) and *B*_*i*_ = *h*(*b*_*i*_). Then, *A* can compute *D*_*i*_ = *h*(*B*_*i*_‖*h*(*x*‖*y*)), *E*_*i*_ = *B*_*i*_ ⊕ *h*(*x*‖*y*), *P*_*ij*_ = *E*_*i*_ ⊕ *h*(*h*(*SID*_*j*_‖*h*(*y*))‖*N*_*i*_), *CID*_*i*_ = *A*_*i*_ ⊕ *h*(*D*_*i*_‖*SID*_*j*_‖*N*_*i*_), *M*_1_ = *h*(*P*_*ij*_‖*CID*_*i*_‖*D*_*i*_‖*N*_*i*_) and *M*_2_ = *h*(*SID*_*j*_‖*h*(*y*)) ⊕ *N*_*i*_ using *h*(*y*) and *h*(*x*‖*y*). Now, *A* sends the login request message {*P*_*ij*_, *CID*_*i*_, *M*_1_, *M*_2_} by masquerading as the user *U*_*i*_ to server *S*_*j*_. After receiving the login request message, *S*_*j*_ computes *N*_*i*_ = *h*(*SID*_*j*_‖*h*(*y*)) ⊕ *M*_2_, *E*_*i*_ = *P*_*ij*_ ⊕ *h*(*h*(*SID*_*j*_‖*h*(*y*))‖*N*_*i*_), *B*_*i*_ = *E*_*i*_ ⊕ *h*(*x*‖*y*), *D*_*i*_ = *h*(*B*_*i*_‖*h*(*x*‖*y*)) and *A*_*i*_ = *CID*_*i*_ ⊕ *h*(*D*_*i*_‖*SID*_*j*_‖*N*_*i*_) using {*P*_*ij*_, *CID*_*i*_, *M*_1_, *M*_2_}, *h*(*x*‖*y*) and *h*(*SID*_*j*_‖*h*(*y*)). Then, *S*_*j*_ computes *M*_3_ = *h*(*D*_*i*_‖*A*_*i*_‖*N*_*j*_‖*SID*_*j*_) and *M*_4_ = *A*_*i*_ ⊕ *N*_*i*_ ⊕ *N*_*j*_ and sends the message {*M*_3_, *M*_4_} to *A*, who is masquerading as the user *U*_*i*_. Then, adversary *A* computes *N*_*j*_ = *A*_*i*_ ⊕ *N*_*i*_ ⊕ *M*_4_ and verifies *M*_3_ by computing *h*(*D*_*i*_‖*A*_*i*_‖*N*_*j*_‖*SID*_*j*_). Then, *A* computes *M*_5_ = *h*(*D*_*i*_‖*A*_*i*_‖*N*_*i*_‖*SID*_*j*_) and sends {*M*_5_} back to the server *S*_*j*_. The server *S*_*j*_ computes *h*(*D*_*i*_‖*A*_*i*_‖*N*_*i*_‖*SID*_*j*_) and checks it against the received message {*M*_5_}. This equivalency authenticates the legitimacy of the user *U*_*i*_ and the service provider server *S*_*j*_, and the login request is accepted. Finally, after mutual authentication, adversary *A* masquerading as the user *U*_*i*_ and the server *S*_*j*_ agree on the common session key as *SK* = *h*(*D*_*i*_‖*A*_*i*_‖*N*_*i*_‖*N*_*j*_‖*SID*_*j*_).

#### Server spoofing attacks

In this subsection, we show that an adversary *A* who possesses *h*(*y*) and *h*(*x*‖*y*) can masquerade as the server *S*_*j*_ to spoof user *U*_*i*_ if *A* has intercepted a valid login request message {*P*_*ij*_, *CID*_*i*_, *M*_1_, *M*_2_} sent from user *U*_*i*_ to server *S*_*j*_ over a public communication channel.

After intercepting a valid login request message {*P*_*ij*_, *CID*_*i*_, *M*_1_, *M*_2_} sent from user *U*_*i*_ to server *S*_*j*_ over a public communication channel, *A* can compute *N*_*i*_ = *h*(*SID*_*j*_‖*h*(*y*)) ⊕ *M*_2_, *E*_*i*_ = *P*_*ij*_ ⊕ *h*(*h*(*SID*_*j*_‖*h*(*y*))‖*N*_*i*_), *B*_*i*_ = *E*_*i*_ ⊕ *h*(*x*‖*y*), *D*_*i*_ = *h*(*B*_*i*_‖*h*(*x*‖*y*)) and *A*_*i*_ = *CID*_*i*_ ⊕ *h*(*D*_*i*_‖*SID*_*j*_‖*N*_*i*_) corresponding to *U*_*i*_. Then, *A* can choose a random number $N_{j}^{\prime }$ and compute $M_{3}=h(D_{i}\|A_{i}\|N_{j}^{\prime }\left\|SID_{j}\right)$ and $M_{4}=A_{i}⊕N_{i}⊕N_{j}^{\prime }$. *A* then sends the message {*M*_3_, *M*_4_} by masquerading as the server *S*_*j*_ to the user *U*_*i*_. After receiving the message {*M*_3_, *M*_4_}, *U*_*i*_ computes $N_{j}^{\prime }=A_{i}⊕N_{i}⊕M_{4}$ and verifies *M*_3_ by computing $h(D_{i}\|A_{i}\|N_{j}^{\prime }\|SID_{j})$. Then, *U*_*i*_ computes *M*_5_ = *h*(*D*_*i*_‖*A*_*i*_‖*N*_*i*_‖*SID*_*j*_) and sends it to the server *S*_*j*_, who is masquerading as the adversary *A*. Then, *A* computes *h*(*D*_*i*_‖*A*_*i*_‖*N*_*i*_‖*SID*_*j*_) and checks it against the received message {*M*_5_}. Finally, after mutual authentication, the adversary *A* masquerading as the server *S*_*j*_ and the user *U*_*i*_ agree on the common session key as $SK=h(D_{i}\|A_{i}\|N_{i}\|N_{j}^{\prime }\|SID_{j})$.

### Discussion

Except for Li et al.’s scheme, we also analyzed four other dynamic ID-based authentication schemes for multi-server environments [15, 17–19]. These schemes are all based on hash functions and are not dependent on RCs. We found that this type of multi-server remote user authentication scheme is generally vulnerable to stolen smart card and offline dictionary attacks, impersonation attacks, server spoofing attacks etc. The cryptanalysis methods used by these schemes are similar to that of Li et al.’s scheme shown in Section 4.2. We believe that under the assumptions that no RC participates in the authentication and session key agreement phase, the dynamic ID and hash function-based user authentication schemes for multi-server environments face difficulties in providing perfectly efficient and secure authentication. Fortunately, there is another technique, public-key cryptography, that is widely used in the construction of authentication schemes. Therefore, to construct a secure, low-power-consumption and non-RC-dependent authentication scheme, we adopt the elliptic curve cryptographic technology of public-key techniques, and we propose a novel dynamic ID-based and non-RC-dependent remote user authentication scheme using pairing and self-certified public keys for multi-server environments.

## The proposed scheme

In this section, we propose a novel dynamic ID-based and non-RC-dependent remote user authentication scheme for multi-server environments using pairing and self-certified public keys. Our scheme contains three participants: the user *U*_*i*_, the service provider server *S*_*j*_, and the registration center *RC*. A legitimate user *U*_*i*_ can easily login to the service provider server using his smart card, identity and password. There are six phases in the proposed scheme: the system initialization phase, the user registration phase, the server registration phase, the login phase, the authentication and session key agreement phase, and the password change phase. The notations used in our proposed scheme are summarized in Table 1.

**Table 1:** Notations used in the proposed scheme.

*e*	A bilinear map, *e*: *G*_1_ × *G*_1_ → *G*_2_.
*U*_*i*_	The *i*th user.
*ID*_*i*_	The identity of the user *U*_*i*_.
*S*_*j*_	The *j*th service provider server.
*SID*_*j*_	The identity of the service provider server *S*_*j*_.
*RC*	The registration center.
*s*_*RC*_	The master secret key of the registration center *RC* in $Z_{q}^{*}$.
*pub*_*RC*_	The public key of *RC*, *pub*_*RC*_ = *s*_*RC*_ ⋅ *P*.
*P*	A generator of group *G*_1_.
*H*()	A map-to-point function, *H*: 0, 1* → *G*_1_.
*h*()	A one-way hash function, *h*: 0, 1* → 0, 1^*k*^, where *k* is the output length. *h*() allows the concatenation of some integer values and points on an elliptic curve.
⊕	A simple XOR operation in *G*_1_. If *P*_1_, *P*_2_ ∈ *G*_1_, *P*_1_ and *P*_2_ are points on an elliptic curve over a finite field, the operation *P*_1_ ⊕ *P*_2_ means that it performs the XOR operations of the x-coordinates and y-coordinates of *P*_1_ and *P*_2_, respectively.
∥	The concatenation operation.

### System initialization phase

In the proposed scheme, the registration center *RC* is assumed to be a TTP. In the system initialization phase, *RC* generates all the needed parameters of the scheme.

(1) The *RC* selects a cyclic additive group *G*_1_ of prime order *q*, a cyclic multiplicative group *G*_2_ of the same order *q*, a generator *P* of *G*_1_, and a bilinear map *e*: *G*_1_ × *G*_1_ → *G*_2_.

(2) The *RC* freely chooses a number $s_{RC}\in Z_{q}^{*}$ held as the system private key and computes *pub*_*RC*_ = *s*_*RC*_ ⋅ *P* as the system public key.

(3) The *RC* selects two cryptographic hash functions *H*(⋅) and *h*(⋅).

Finally, all the related parameters {*e*, *G*_1_, *G*_2_, *q*, *P*, *Pub*_*RC*_, *H*(⋅), *h*(⋅)} are publicly and authentically available.

### User registration phase

When the user *U*_*i*_ wants to access the services, he/she has to submit some of his/her related information to the registration center *RC* for registration. The steps of the user registration phase are as follows:

(1) *U*_*i*_ freely generates his/her identity *ID*_*i*_ and password *pw*_*i*_ and chooses a random number *b*_*i*_. Then, *U*_*i*_ computes *HPW*_*i*_ = *h*(*ID*_*i*_ ∥ *pw*_*i*_ ∥ *b*_*i*_) ⋅ *P* and submits *ID*_*i*_ and *HPW*_*i*_ to *RC* for registration through a secure channel.

(2) When receiving the message *ID*_*i*_ and *HPW*_*i*_, *RC* computes *QID*_*i*_ = *H*(*ID*_*i*_), *CID*_*i*_ = *s*_*RC*_ ⋅ *QID*_*i*_, $Reg_{ID_{i}}=CID_{i}⊕s_{RC}\cdot HPW_{i}$ and *H*_*i*_ = *h*(*QID*_*i*_ ∥ *CID*_*i*_). Then, *RC* stores the message $\left\{Reg_{ID_{i}},H_{i}\right\}$ in *U*_*i*_’s smart card and submits the smart card to *U*_*i*_ through a secure channel.

(3) After receiving the smart card, *U*_*i*_ enters *b*_*i*_ into the smart card. Finally, the smart card contains the parameters $\left\{Reg_{ID_{i}},H_{i},b_{i}\right\}$.

### Server registration phase

If a service provider server *S*_*j*_ wants to provide services to the users, he/she must perform the registration to the registration center *RC* to become a legal service provider server. The process of the server registration phase of the proposed scheme is based on SCPK.

(1) *S*_*j*_ chooses a random number *v*_*j*_ and computes *V*_*j*_ = *v*_*j*_ ⋅ *P*. Then, *S*_*j*_ submits *SID*_*j*_ and *V*_*j*_ to *RC* for registration via a secure channel.

(2) After receiving the message {*SID*_*j*_, *V*_*j*_}, *RC* chooses a random number *w*_*j*_ and computes *W*_*j*_ = *w*_*j*_ ⋅ *P* + *V*_*j*_ and $s_{j}^{\prime }=\left(s_{RC}\cdot h\left(SID_{j}\|W_{j}\right)+w_{j}\right)$ mod *q*. Then, *RC* submits the message $\left\{W_{j},s_{j}^{\prime }\right\}$ to *S*_*j*_ through a secure channel.

(3) After receiving $\left\{W_{j},s_{j}^{\prime }\right\}$, *S*_*j*_ computes their private key $s_{j}=\left(s_{j}^{\prime }+v_{j}\right)$ mod *q* and checks the validity of the values issued to them by checking the following equation: *pub*_*j*_ = *s*_*j*_ ⋅ *P* = *h*(*SID*_*j*_ ∥ *W*_*j*_) ⋅ *pub*_*RC*_ + *W*_*j*_. Finally, *S*_*j*_’s personal information contains {*SID*_*j*_, *pub*_*j*_, *s*_*j*_, *W*_*j*_}

**Fig 1:**
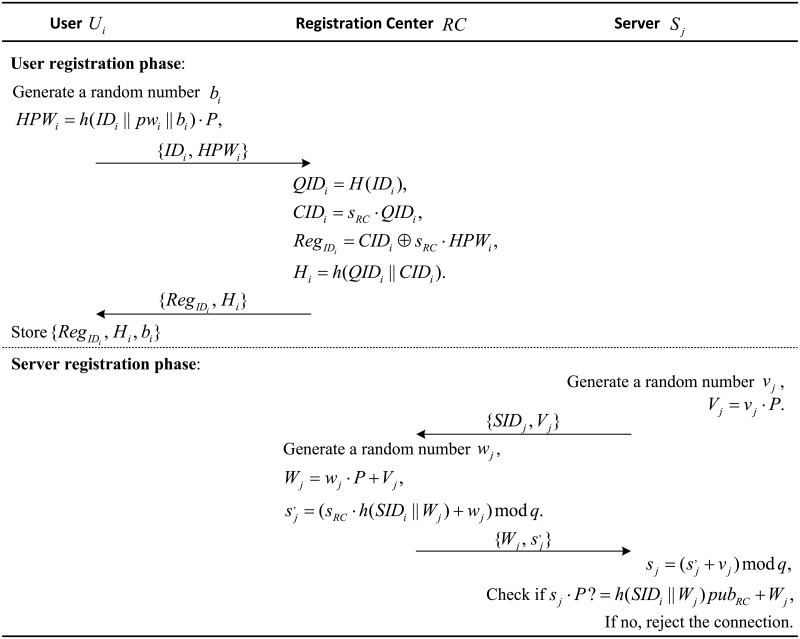
User and server registration phases of the proposed scheme.

The details of the user registration phase and server registration phase are shown in Fig 1.

### Login phase

If user *U*_*i*_ wants to access the services provided by server *S*_*j*_, *U*_*i*_ needs to login to *S*_*j*_, where the process of the login phase are as follows:

(1) The user *U*_*i*_ inserts their smart card into the smart card reader and inputs their identity *ID*_*i*_ and password *pw*_*i*_. The smart card then calculates *QID*_*i*_ = *H*(*ID*_*i*_), $CID_{i}=Reg_{ID_{i}}⊕h\left(ID_{i}\|pw_{i}\|b_{i}\right)\cdot pub_{RC}$, and $H_{i}^{*}=h\left(QID_{i}\|CID_{i}\right)$ and verifies whether $H_{i}^{*}$ is equal to *H*_*i*_. If they are equal, it is verified that *U*_*i*_ has the correct user identity and password. Thus, *U*_*i*_ is a legitimate user. Otherwise, the smart card aborts the session.

(2) The smart card chooses two random numbers *u*_*i*_ and *r*_*i*_, and it computes *DID*_*i*_ = *u*_*i*_ ⋅ *QID*_*i*_ and *R*_*i*_ = *r*_*i*_ ⋅ *P*. Then, the smart card sends the login request message {*DID*_*i*_, *R*_*i*_} to server *S*_*j*_ over a public channel.

### Authentication and session key agreement phase

(1) Based on the received login request message {*DID*_*i*_, *R*_*i*_} sent from the user *U*_*i*_, the server *S*_*j*_ chooses a random number *r*_*j*_ and computes *R*_*j*_ = *r*_*j*_ ⋅ *P*, *T*_*ji*_ = *r*_*j*_ ⋅ *R*_*i*_, *K*_*ji*_ = *s*_*j*_ ⋅ *R*_*i*_ and *Auth*_*ji*_ = *h*(*DID*_*i*_ ∥ *SID*_*j*_ ∥ *K*_*ji*_ ∥ *R*_*j*_). Then, *S*_*j*_ sends the message {*W*_*j*_, *R*_*j*_, *Auth*_*ji*_} to *U*_*i*_.

(2) When receiving {*W*_*j*_, *R*_*j*_, *Auth*_*ji*_}, *U*_*i*_ computes *T*_*ij*_ = *r*_*i*_ ⋅ *R*_*j*_, *pub*_*j*_ = *h*(*SID*_*j*_ ∥ *W*_*j*_) ⋅ *pub*_*RC*_ + *W*_*j*_, *K*_*ij*_ = *r*_*i*_ ⋅ *pub*_*j*_ and *Auth*_*ij*_ = *h*(*DID*_*i*_ ∥ *SID*_*j*_ ∥ *K*_*ij*_ ∥ *R*_*j*_). Then, *U*_*i*_ checks *Auth*_*ij*_ with the received *Auth*_*ji*_. If they are not equal, *U*_*i*_ terminates this session. Otherwise, *S*_*j*_ is proven to have the correct private key *s*_*j*_, and thus, *S*_*j*_ is authenticated. *U*_*i*_ continues to compute *M*_*i*_ = *r*_*i*_ ⋅ *DID*_*i*_, *N*_*i*_ = *u*_*i*_ ⋅ *CID*_*i*_, *d*_*ij*_ = *h*(*DID*_*i*_ ∥ *SID*_*j*_ ∥ *K*_*ij*_ ∥ *M*_*i*_) and *B*_*i*_ = (*r*_*i*_ + *d*_*ij*_) ⋅ *N*_*i*_. Finally, *U*_*i*_ sends the message {*M*_*i*_, *B*_*i*_} to *S*_*j*_.

(3) After receiving the message {*M*_*i*_, *B*_*i*_} sent from *U*_*i*_, *S*_*j*_ computes *d*_*ji*_ = *h*(*DID*_*i*_ ∥ *SID*_*j*_ ∥ *K*_*ji*_ ∥ *M*_*i*_) and checks whether *e*(*M*_*i*_ + *d*_*ji*_ ⋅ *DID*_*i*_, *pub*_*RC*_) = *e*(*B*_*i*_, *P*). If they are not equal, *S*_*j*_ terminates this session. Otherwise, *U*_*i*_ is authenticated.

Finally, the user *U*_*i*_ and the server *S*_*j*_ agree on a common session key as *U*_*i*_: *SK* = *h*(*DID*_*i*_ ∥ *SID*_*j*_ ∥ *K*_*ij*_ ∥ *T*_*ij*_), *S*_*j*_: *SK* = *h*(*DID*_*i*_ ∥ *SID*_*j*_ ∥ *K*_*ji*_ ∥ *T*_*ji*_).

**Fig 2:**
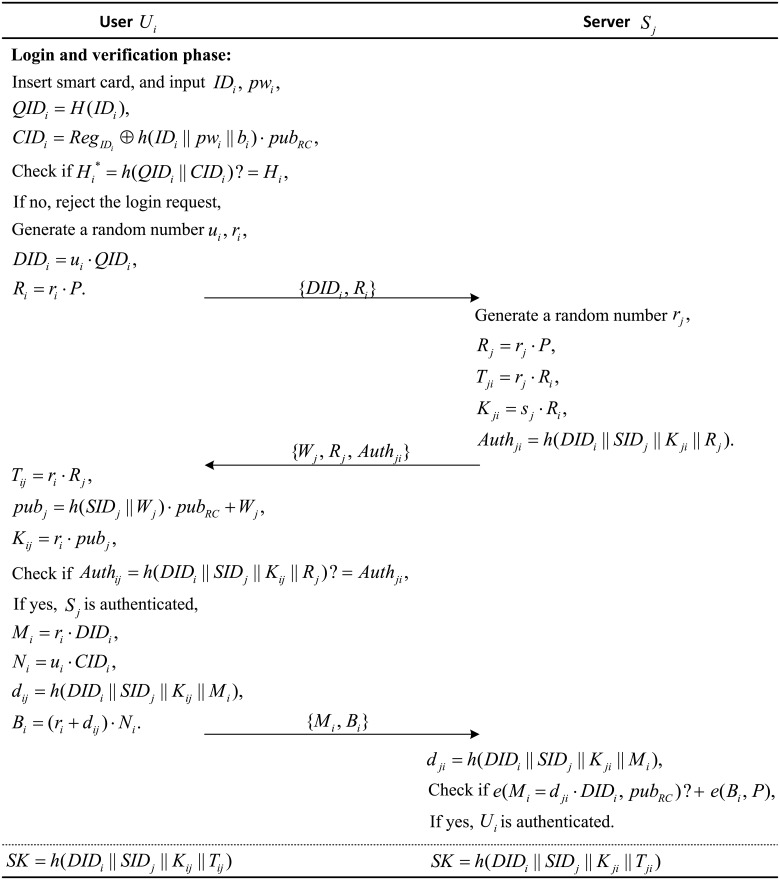
Login phase and authentication and session key agreement phase.

Sections 5.4 and 5.5 give the detailed procedures of the login phase and authentication and session key agreement phase, which are also depicted in Fig 2.

### Password change phase

For security purposes, users need to change their passwords frequently. The following steps show the password change phase process for a user *U*_*i*_.

(1) The user *U*_*i*_ inserts his/her smart card into the smart card reader and inputs their identity *ID*_*i*_ and password *pw*_*i*_. Then, the smart card computes *QID*_*i*_ = *H*(*ID*_*i*_), $CID_{i}=Reg_{ID_{i}}⊕h\left(ID_{i}\|pw_{i}\|b_{i}\right)\cdot pub_{RC}$, $H_{i}^{*}=h\left(QID_{i}\|CID_{i}\right)$ and checks whether $H_{i}^{*}=H_{i}$. If they are equal, *U*_*i*_ is verified as a legitimate user; otherwise, the smart card rejects the password change request.

(2) The smart card generates a random number *z*_*i*_ and computes *Z*_*i*_ = *z*_*i*_ ⋅ *P* and *AID*_*i*_ = *CID*_*i*_ ⊕ *z*_*i*_ ⋅ *pub*_*RC*_. Then, the smart card sends the message {*ID*_*i*_, *AID*_*i*_, *Z*_*i*_} to the registration center *RC*.

(3) After receiving the message {*ID*_*i*_, *AID*_*i*_, *Z*_*i*_}, *RC* computes *CID*_*i*_ = *AID*_*i*_ ⊕ *s*_*RC*_ ⋅ *Z*_*i*_, *QID*_*i*_ = *H*(*ID*_*i*_), and checks whether *e*(*CID*_*i*_, *P*) = *e*(*QID*_*i*_, *pub*_*RC*_). If they are equal, user *U*_*i*_ is authenticated. Then, *RC* computes *V*_1_ = *h*(*CID*_*i*_ ∥ *s*_*RC*_ ⋅ *Z*_*i*_) and sends {*V*_1_} to *U*_*i*_.

(4) When receiving {*V*_1_}, the user computes $V_{1}^{*}=h\left(CID_{i}\|z_{i}\cdot pub_{RC}\right)$ and checks it against the received *V*_1_. If they are equal, the registration center *RC* is authenticated. Then, *U*_*i*_ chooses his/her new password $pw_{i}^{new}$ and the new random number $b_{i}^{new}$, and they compute $HPW_{i}^{new}=h\left(ID_{i}\|pw_{i}^{new}\|b_{i}^{new}\right)\cdot P$, $V_{2}=HPW_{i}^{new}⊕z_{i}\cdot pub_{RC}$ and $V_{3}=h\left(CID_{i}\|z_{i}\cdot pub_{RC}\|HPW_{i}^{new}\right)$. Then, *U*_*i*_ submits {*V*_2_, *V*_3_} to *RC*.

(5) Upon receiving the response {*V*_2_, *V*_3_}, the registration server *RC* computes $HPW_{i}^{new}=V_{2}⊕s_{RC}\cdot Z_{i}$ and $V_{3}^{*}=h\left(CID_{i}\|s_{RC}\cdot Z_{i}\|HPW_{i}^{new}\right)$. Then, *RC* compares $V_{3}^{*}$ with the received *V*_3_. If they are equal, *RC* continues to compute $Reg_{ID_{i}}^{new}=CID_{i}⊕s_{RC}\cdot HPW_{i}^{new}$, $V_{4}=Reg_{ID_{i}}^{new}⊕s_{RC}\cdot Z_{i}$ and $V_{5}=h\left(s_{RC}\cdot Z_{i}\|Reg_{ID_{i}}^{new}\right)$. After that, *RC* sends {*V*_4_, *V*_5_} to *U*_*i*_.

(6) After receiving {*V*_4_, *V*_5_}, *U*_*i*_ computes $Reg_{ID_{i}}^{new}=V_{4}⊕z_{i}\cdot pub_{RC}$ and $V_{5}^{*}=h\left(z_{i}\cdot pub_{RC}\|Reg_{ID_{i}}^{new}\right)$. Then, *U*_*i*_ checks whether $V_{5}^{*}=V_{5}$. If they are equal, user *U*_*i*_ replaces the original $Reg_{ID_{i}}$ and *b*_*i*_ with $Reg_{ID_{i}}^{new}$ and $b_{i}^{new}$.

**Fig 3:**
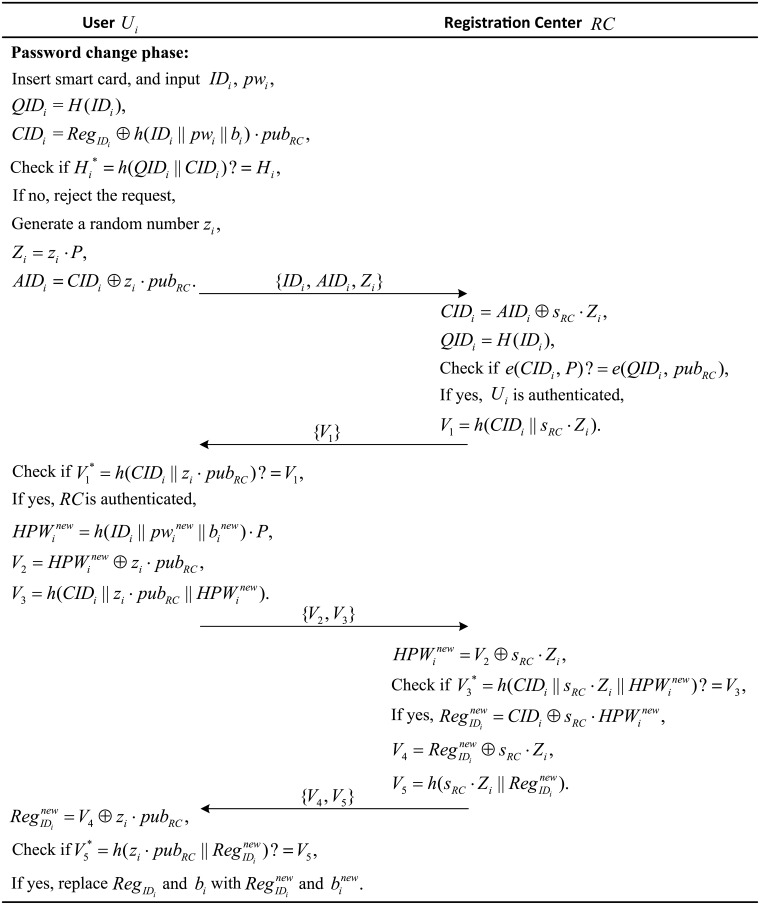
Password change phase of the proposed scheme.

In addition to the descriptions listed above, the procedures of the password change phase of the proposed scheme are also given in Fig 3.

## Security analysis

### Stolen smart card and offline dictionary attacks

In the proposed scheme, we assume that if a smart card is stolen, physical protection methods cannot prevent malicious attackers for obtaining the stored secure elements. Simultaneously, an adversary *A* can access a large dictionary of words that likely includes the user’s password and intercept the communications between the user and server.

In the proposed scheme, if a user *U*_*i*_’s smart card is stolen by an adversary *A*, the latter can extract $\left\{Reg_{ID_{i}},H_{i},b_{i}\right\}$ from the memory of the stolen smart card. Simultaneously, it is assumed that adversary *A* has intercepted a previous full session of messages {*DID*_*i*_, *R*_*i*_, *W*_*j*_, *R*_*j*_, *Auth*_*ji*_, *M*_*i*_, *B*_*i*_} between the user *U*_*i*_ and server *S*_*j*_. However, the adversary still cannot obtain *U*_*i*_’s identity *ID*_*i*_ and password *pw*_*i*_ except by guessing *ID*_*i*_ and *pw*_*i*_ simultaneously. Therefore, it is impossible to obtain *U*_*i*_’s identity *ID*_*i*_ and password *pw*_*i*_ from a stolen smart card and using offline dictionary attacks in our proposed scheme.

### Replay attacks

Replaying a message of a previous session into a new session is useless in our proposed scheme because the user’s smart card and the server choose different rand numbers *r*_*i*_ and *r*_*j*_, and the user’s identity is different in each new session. These factors make all messages dynamic and valid for that session only. If we assume that an adversary *A* replies with an intercepted previous login request {*DID*_*i*_, *R*_*i*_} to *S*_*j*_, after receiving the response message {*W*_*j*_, *R*_*j*_, *Auth*_*ji*_} sent from *S*_*j*_, *A* cannot compute the correct response message {*M*_*i*_, *B*_*i*_} to pass *S*_*j*_’s authentication since they do not know the values of *ID*_*i*_, *pw*_*i*_, *u*_*i*_ and *r*_*i*_. Therefore, the proposed scheme is robust to replay attacks.

### Impersonation attacks

If an adversary *A* wants to masquerade as a legitimate user *U*_*i*_ to pass the authentication of a server *S*_*j*_, the user must have the values of both *QID*_*i*_ and *CID*_*i*_. However, *QID*_*i*_ and *CID*_*i*_ are protected by *U*_*i*_’s smart card, *ID*_*i*_ and *pw*_*i*_ since *QID*_*i*_ = *H*(*ID*_*i*_) and $CID_{i}=Reg_{ID_{i}}⊕h\left(ID_{i}\|pw_{i}\|b_{i}\right)\cdot pub_{RC}$. Therefore, unless the adversary *A* can obtain the user *U*_*i*_’s smart card, *ID*_*i*_ and *pw*_*i*_ simultaneously, the proposed scheme is secure to impersonation attacks.

### Server spoofing attacks

If an adversary *A* wants to masquerade as a legal server *S*_*j*_ to cheat a user *U*_*i*_, the adversary must calculate a valid *Auth*_*ji*_ that is embedded with the shared secret key *K*_*ji*_ = *s*_*j*_ ⋅ *R*_*i*_ to pass the authentication of *U*_*i*_. However, the adversary *A* cannot derive the shared secret key *K*_*ji*_ without knowing the private key *s*_*j*_ of the server *S*_*j*_. Therefore, our scheme is secure against server spoofing attacks.

### Insider attacks

In the proposed scheme, the registration center *RC* cannot obtain *U*_*i*_’s password *pw*_*i*_. Since in the registration phase *U*_*i*_ chooses a random number *b*_*i*_ and sends *ID*_*i*_ and *HPW*_*i*_ = *h*(*ID*_*i*_ ∥ *pw*_*i*_ ∥ *b*_*i*_) ⋅ *P* to *RC*, *RC* cannot derive *pw*_*i*_ from *HPW*_*i*_ based on the CDL problem. Therefore, the proposed scheme is robust to insider attacks.

### Denial of service attacks

In denial of service attacks, an adversary *A* updates the identity and password verification information on the smart card to some arbitrary value, and hence, legitimate users cannot login successfully in subsequent login requests to the server. In the proposed scheme, the smart card checks the validity of user *U*_*i*_’s identity *ID*_*i*_ and password *pw*_*i*_ before the password update procedure. An adversary can insert the stolen smart card of the user *U*_*i*_ into the smart card reader and must guess the identity *ID*_*i*_ and password *pw*_*i*_ corresponding to the user *U*_*i*_ correctly. The smart card computes $H_{i}^{*}=h\left(QID_{i}\|CID_{i}\right)$ and compares it with the stored value of *H*_*i*_ in its memory to verify the legitimacy of the user *U*_*i*_ before the smart card accepts the password update request. It is not possible to guess the identity *ID*_*i*_ and password *pw*_*i*_ correctly simultaneously in real polynomial time even after obtaining the smart card of the user *U*_*i*_. Therefore, the proposed scheme is secure against denial of service attacks.

### Perfect forwarding secrecy

Perfect forwarding secrecy means that even if an adversary compromises all the passwords of the users, it still cannot compromise the session key. In the proposed scheme, the session key *SK* = *h*(*DID*_*i*_ ∥ *SID*_*j*_ ∥ *K*_*ij*_ ∥ *T*_*ij*_) *SK* = *h*(*DID*_*i*_ ∥ *SID*_*j*_ ∥ *K*_*ij*_ ∥ *T*_*ji*_) is generated by three single-use random numbers *u*_*i*_, *r*_*i*_ and *r*_*j*_ in each session. These single-use random numbers are only held by the user *U*_*i*_ and the server *S*_*j*_ and cannot be retrieved from *SK* based on the security of the CDH problem. Thus, even if an adversary obtains previous session keys, it cannot compromise other session keys. Hence, the proposed scheme achieves perfect forwarding secrecy.

### User anonymity

In our proposed scheme, the user *U*_*i*_’s login message is different in each login phase. For each login message, *DID*_*i*_ = *u*_*i*_ ⋅ *H*(*ID*_*i*_) is associated with a random number *u*_*i*_, which is known by *U*_*i*_ alone. Therefore, no adversary can identity the real identity of the logged on user, and our scheme can ensure the user’s anonymity.

### No verification table

In our proposed scheme, it is obvious that the user, server and registration center do not maintain a verification table.

### Local password verification

In the proposed scheme, the smart card checks the validity of user *U*_*i*_’s identity *ID*_*i*_ and password *pw*_*i*_ before logging into server *S*_*j*_. Since the adversary cannot compute the correct *CID*_*i*_ without knowledge of *ID*_*i*_ and *pw*_*i*_ to satisfy the verification equation $H_{i}^{*}=H_{i}$, our scheme can avoid unauthorized access via local password verification.

### Proper mutual authentication

In our scheme, the user first authenticates the server. *U*_*i*_ sends the message {*DID*_*i*_, *R*_*i*_} to the server *S*_*j*_ to establish a connection. After receiving the response message {*W*_*j*_, *R*_*j*_, *Auth*_*ji*_} sent from *S*_*j*_, *U*_*i*_ computes *T*_*ij*_, *pub*_*j*_, *K*_*ij*_, and *Auth*_*ij*_ and checks whether *Auth*_*ij*_ = *Auth*_*ji*_. If they are equal, *S*_*j*_ is authenticated by *U*_*i*_. Otherwise, *U*_*i*_ stops to login to this server. Since *Auth*_*ji*_ = *h*(*DID*_*i*_ ∥ *SID*_*j*_ ∥ *K*_*ji*_ ∥ *R*_*j*_) and *K*_*ji*_ = *s*_*j*_ ⋅ *R*_*i*_, an adversary *A* cannot compute the correct *K*_*ji*_ without knowledge of the value of *s*_*j*_. Any fabricated message $\left\{W_{j}^{\prime },R_{j}^{\prime },Auth_{ji}^{\prime }\right\}$ cannot pass verification. Then, *U*_*i*_ computes *M*_*i*_, *N*_*i*_, *d*_*ij*_, and *B*_*i*_ and sends the message {*M*_*i*_, *B*_*i*_} to *S*_*j*_. After receiving the message {*M*_*i*_, *B*_*i*_} sent from *U*_*i*_, *S*_*j*_ computes *d*_*ji*_ and checks whether *e*(*M*_*i*_ + *d*_*ji*_ ⋅ *DID*_*i*_, *pub*_*RC*_) = *e*(*B*_*i*_, *P*). If they are not equal, *S*_*j*_ terminates this session; otherwise, *U*_*i*_ is authenticated. Since *B*_*i*_ = (*r*_*i*_ + *d*_*ij*_) ⋅ *N*_*i*_, an adversary *A* cannot compute the correct *B*_*i*_ without knowledge of the values of *u*_*i*_, *r*_*i*_ etc. Any fabricated message $\left\{M_{i}^{\prime },B_{i}^{\prime }\right\}$ cannot pass verification. Therefore, our proposed scheme can provide proper mutual authentication.

## Performance comparison and functionality analysis

In this section, we compare the performance and functionality of our proposed scheme with some previous schemes. To analyze the computation cost, some notations are defined as follows.

*TG*_*e*_: The time for executing a bilinear map operation, *e*: *G*_1_ × *G*_1_ → *G*_2_.
*TG*_*mul*_: The time for executing point scalar multiplication on the group *G*_1_.
*TG*_*H*_: The time for executing a map-to-point hash function H(.).
*TG*_*add*_: The time for executing point addition on the group *G*_1_.
*T*_*h*_: The time for executing a one-way hash function *h*(.).

Since the XOR operation and the modular multiplication operation require very few computations, it is usually negligible considering their computation costs.

Table 2 shows the performance comparisons of our proposed scheme and various other related protocols. We focus on three computational costs: C1, the total time for all operations executed during the user registration phase; C2, the total time spent by the user during the login phase and verification phase; and C3, the total time spent by the server during the verification phase. As shown in Table 2, Tseng et al.’s scheme is more efficient in terms of computational cost. However, Tseng et al.’s scheme is vulnerable to stolen smart card and offline dictionary attacks, server spoofing attacks and insider attacks and cannot provide perfect forwarding secrecy, user anonymity, proper mutual authentication and session key agreement. In our proposed scheme, the total computational cost for the user (C2) is 9*TG*_*mul*_+*TG*_*H*_+*TG*_*add*_+5*T*_*h*_. However, similar to Liao et al.’s scheme, the user *U*_*i*_ can pre-compute *R*_*i*_ = *r*_*i*_ ⋅ *P* in the client, and then, the computational cost of the user (C2) requires 8*TG*_*mul*_+*TG*_*H*_+*TG*_*add*_+5*T*_*h*_ on-line computations. It can be found that our proposed scheme has a slightly higher computational cost than Liao et al.’s scheme in C2, and the others are almost equal. However, Liao et al.’s scheme is vulnerable to stolen smart card and offline dictionary attacks and denial of service attacks and cannot provide user anonymity and local password verification.

**Table 2:** Computational cost comparison of our scheme with other schemes.

	Proposed scheme	Liao et al.’scheme [27]	Tseng et al.’scheme [26]
C1	3*TG*_*mul*_+*TG*_*H*_+2*T*_*h*_	3*TG*_*mul*_+*TG*_*H*_+*T*_*h*_	2*TG*_*mul*_+*TG*_*H*_+*T*_*h*_
C2	8*TG*_*mul*_+*TG*_*H*_+*TG*_*add*_+5*T*_*h*_	5*TG*_*mul*_+*TG*_*H*_+*TG*_*add*_+5*T*_*h*_	3*TG*_*mul*_+2*T*_*h*_
C3	2*TG*_*e*_+4*TG*_*mul*_+*TG*_*add*_+2*T*_*h*_	2*TG*_*e*_+5*TG*_*mul*_+*TG*_*add*_+2*T*_*h*_	2*TG*_*e*_+*TG*_*mul*_+*TG*_*H*_+*TG*_*add*_+*T*_*h*_

Table 3 lists the functionality comparisons among our proposed scheme and other related schemes. It is obvious that our scheme has many excellent features and is more secure than other related schemes.

**Table 3:** Functionality comparisons among related multi-server authentication protocols.

	Proposed scheme	Liao et al. [27]	Tseng et al. [26]	Li et al. [20]	Lee et al. [18]	Shao et al. [17]	Lee et al. [19]
Resist stolen smart card and offline dictionary attacks	Yes	No	No	No	No	No	No
Resist replay attacks	Yes	Yes	Yes	No	No	No	No
Resist impersonation attacks	Yes	Yes	Yes	No	No	No	No
Resist server spoofing attacks	Yes	Yes	No	No	No	No	No
Resist insider attacks	Yes	Yes	No	Yes	Yes	No	Yes
Resist denial of service attacks	Yes	No	Yes	Yes	Yes	Yes	No
Perfect forwarding secrecy	Yes	Yes	No	Yes	Yes	No	No
Ensure user’s anonymity	Yes	No	No	Yes	Yes	No	Yes
No verification table	Yes	Yes	Yes	Yes	Yes	Yes	Yes
Local password verification	Yes	No	Yes	Yes	Yes	Yes	No
Proper mutual authentication	Yes	Yes	No	Yes	No	Yes	Yes

## Conclusion

In this paper, we note that Li et al.’s scheme is vulnerable to stolen smart card and offline dictionary attacks, replay attacks, impersonation attacks and server spoofing attacks. Furthermore, by analyzing some other similar schemes, we find that certain types of dynamic ID-based and non-RC-dependent multi-server authentication schemes in which only hash functions are used face difficulties in providing perfectly efficient and secure authentication. To compensate for these shortcomings, we propose a novel dynamic ID-based and non-RC-dependent remote user authentication scheme for multi-server environments using pairing and self-certified public keys. The security and performance analyses show that the proposed scheme is secure against various attacks and has many excellent features. In the future, the use of authentication for high-tech industries, such as cloud computing [42–44] and big data [44–46], will be an important area and research task.
